# Identification of X Monosomy Cells From a Gonad of Mixed Gonadal Dysgenesis With a 46,XY Karyotype: Case Report

**DOI:** 10.1097/MD.0000000000000720

**Published:** 2015-04-10

**Authors:** Noriko Nishina-Uchida, Ryuji Fukuzawa, Yukihiro Hasegawa, Ian M. Morison

**Affiliations:** From the Molecular and Developmental Pathology Research Group (NN-U, RF); Division of Endocrinology and Metabolism (NN-U, YH); Department of Pathology and Laboratory Medicine, Tokyo Metropolitan Children's Medical Center, 2-8-29 Musashidai, Fuchu, Tokyo, Japan (RF); and Department of Pathology, Dunedin School of Medicine, University of Otago, P.O. Box 913, Dunedin, New Zealand (RF, IMM).

## Abstract

Mixed gonadal dysgenesis (MGD) is a disorder of sexual development that typically has a mosaic 45,X/46,XY karyotype.

A 1-year-old infant with 46,XY identified by peripheral blood karyotype demonstrated clinical manifestations and gonadal pathologic features of MGD. Fluorescence in situ hybridization (FISH) for X and Y chromosomes and immunofluorescence for SRY along with testicular and ovarian lineage markers SOX9 and FOXL2, respectively, were performed on paraffin sections from the gonad to ascertain the somatic mosaic state for 45,X monosomy and 46,XY cells. The gonad consisted of cells with X and XY signals, which were further quantified in comparison with a normal control testis by a digital image analysis program.

The average percentages of 45,X cells of this patient's gonad and a control testis were 39.0% and 5.7%, respectively (χ^2^ test, *P* < 0.001). SRY expression was absent in approximately 10% of precursor granulosa cells (FOXL2 positive) and precursor Sertoli/granulosa cells (both SOX9 and FOXL2 positive) within gonadoblastomas, confirming the involvement of 45,X cells.

A combination of analysis of FISH and immunofluorescence for SRY in the gonadal tissue could identify 45,X cells in MGD with 46,XY.

## Abstract 2

Supplemental Digital Content is available in the text

## INTRODUCTION

Mixed gonadal dysgenesis (MGD) is a disorder of sexual development that is defined by the presence of a streak gonad on one side and a partial or normal testis on the other.^1^ Patients with MGD have a broad range of external genitalia phenotypes that include normal male, ambiguous, and normal-appearing female.^2^ The peripheral blood of MGD patients typically has a 45,X/46,XY karyotype, which confirms the diagnosis of this disease.^3^ However, it has been reported that a subset of MGD patients have 46,XY.^4^ In those patients with MGD, peripheral blood karyotypes do not reflect the true clinical state,^5^ indicating that the karyotypes from the peripheral blood are inconsistent with those from the gonadal tissue. Taking such cases into consideration, peripheral blood karyotypes are not always sufficient for the diagnosis of MGD. So far, a number of cases have been shown to have a 45,X karyotype in paraffin sections from their gonads.^4–7^ However, it has not been objectively ascertained whether those 45,X cells were derived from a true monosomy cell or were a result of analytical artifacts.

## PATIENT AND METHODS

### Case Study

**FIGURE 1:**
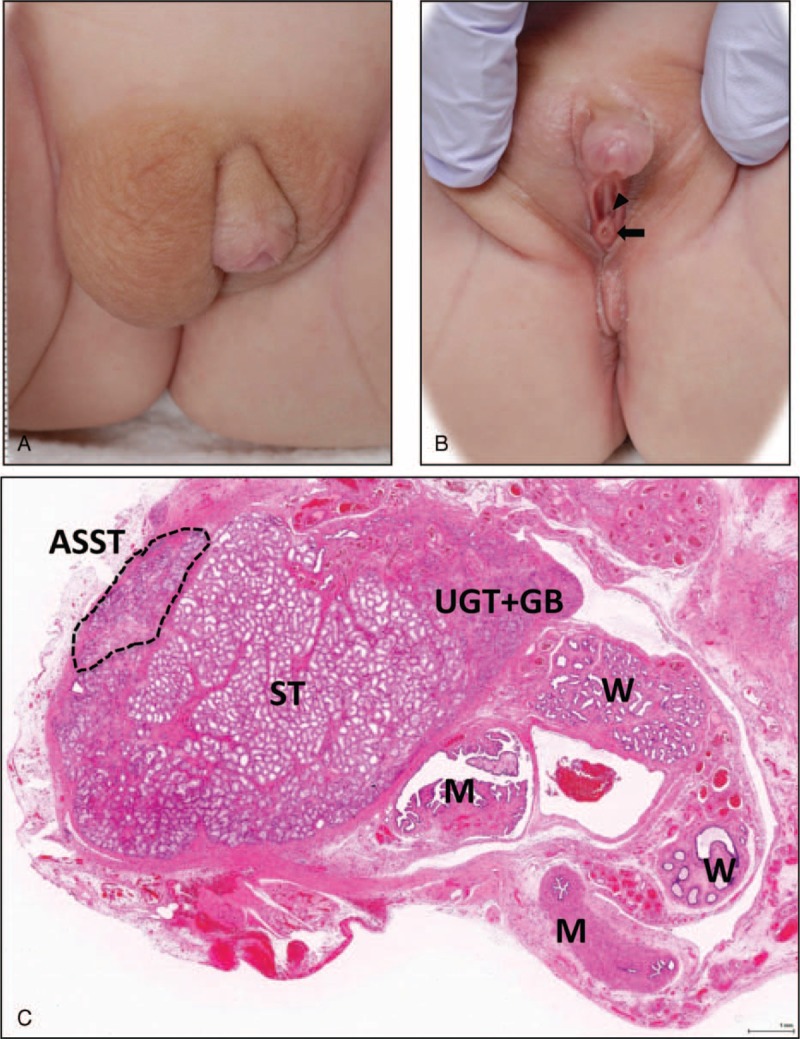
Gross appearance of external genitalia and histology of the right gonad. (A) and (B) Photographs show ambiguous genitalia with clitorimegaly and the separated openings of the external urethra (indicated by an arrowhead) and vagina (indicated by an arrow). Those positional relationships were similar to those of normal females rather than males, thus indicating incomplete virilization of the external genitalia. The asymmetry of the major labia reflects the presence of the palpable right gonad. (C) A scanned image of a longitudinally bisected gonad consists of normal appearing seminiferous tubules (ST), abnormally shaped seminiferous tubule (ASST surrounded by broken lines), an undifferentiated gonadal tissue (UGT), gonadoblastomas (GB), and Wolffian duct (W) and Müllerian duct (M) derivatives.

**FIGURE 2:**
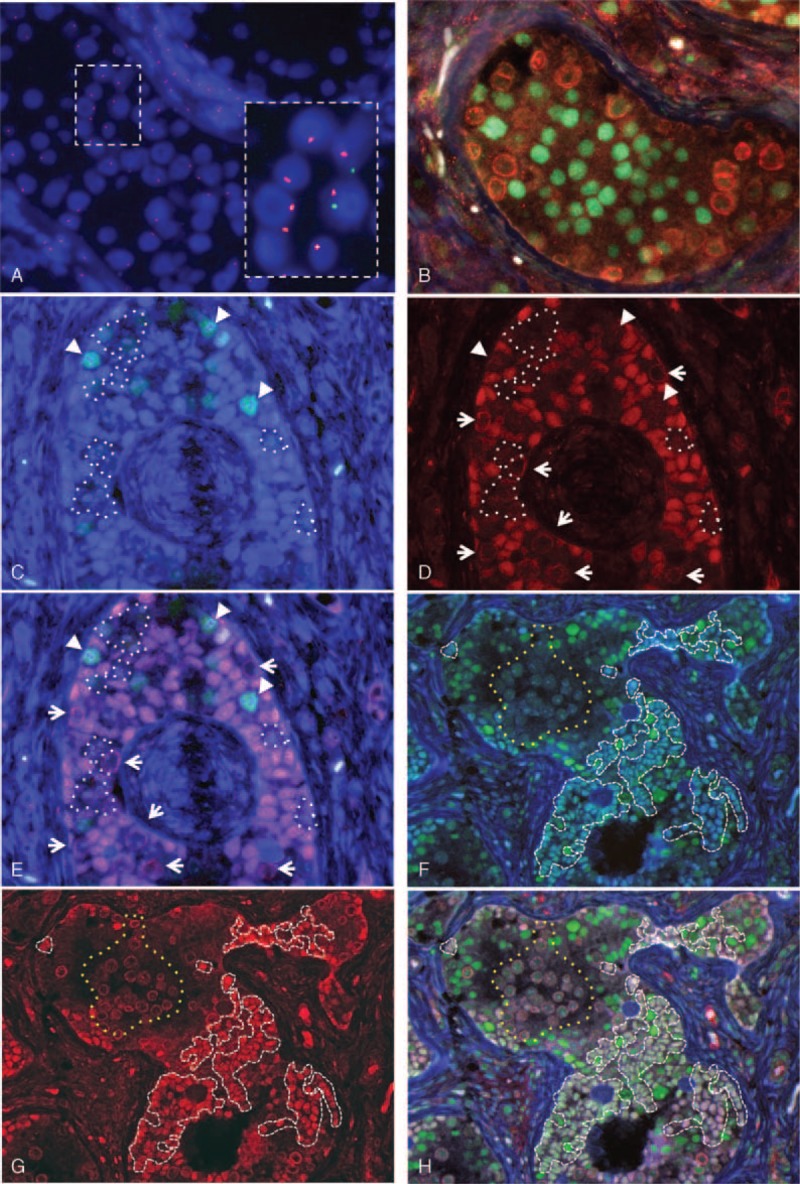
FISH and immunofluorescence analyses of the gonad. (A) The gonadal tissue is composed of 45,X and 46,XY cells. The inset highlights the presence of 45,X cells. Red and green signals denote X and Y probes, respectively. (B) Triple color immunofluorescence for SOX9 (testicular lineage marker: green), SRY (Y-chromosome marker: red), and FOXL2 (ovarian lineage marker: light blue) in a normal appearing seminiferous tubule, consisting of Sertoli cells (nuclear SOX9 signals) and germ cells (membranous/cytoplasmic SRY signals). FOXL2-positive cells are absent. (C) Dual color immunofluorescence for SOX9 (green) and FOXL2 (light blue) in a gonadoblastoma comprising pre-granulosa cells only expressing FOXL2 (light blue nuclei) and pre-Sertoli/granulosa cells expressing both SOX9 and FOXL2 (nuclear emerald green signals indicated by arrowheads). (D) Immunofluorescence for SRY (red) showing that nuclear SRY is present in most pre-granulosa cells, and absent in a minority of pre-granulosa cells (surrounded by white dotted lines). Pre-Sertoli/granulosa cells expressing both SOX9 and FOXL2 (see C) are also devoid of nuclear SRY signals (indicated by arrowheads in C, D, and E). These confirm the presence of 45,X cells. The membranous/cytoplasmic SRY signals indicate the localization of germ cells (indicated by arrows). (E) Triple color immunofluorescence: (C) SOX9 and FOXL2 merged with (D) SRY. The predominant population of sex cord epithelial cells expresses both FOXL2 and SRY (purple nuclei) indicating pre-granulosa cells with a 46,XY karyotype. It is conceivable that a minority of pre-granulosa cells have a 45,X karyotype (surrounded by white dotted lines) because of lack of SRY expression. Some pre-Sertoli/granulosa cells also have a 45,X karyotype (arrow heads), because they express both SOX9 and FOXL2 but lack SRY expression (nuclear emerald green signals). (F) Dual color immunofluorescence for SOX9 and FOXL2 demonstrating abnormally shaped seminiferous tubules composed of mature Sertoli cells only expressing SOX9 (green nuclei) and pre-Sertoli/granulosa cells expressing both SOX9 and FOXL2 (emerald-green nuclei surrounded by white dashed lines). An aggregate of germ cells is surrounded by a yellow dotted line. (G) Immunofluorescence for SRY reveals that the cells expressing FOXL2 have nuclear SRY positivity (red nuclear signals surrounded by white dashed lines). The membranous/cytoplasmic SRY immunoreactivity clarifies an aggregate of germ cells (surrounded by yellow dotted lines). (H) Triple color immunofluorescence: merged SOX9 and FOXL2 (F) with SRY (G). The presence of Sertoli cells with SOX9 expression (green nuclei) and co-localization of SRY signals in the nuclei of pre-Sertoli/granulosa cells with both SOX9 and FOXL2 expression (nuclear white, pink or light green nuclei surrounded by white dashed lines) indicate that the abnormally shaped seminiferous tubule is a male structure, showing incomplete testicular differentiation. FISH = fluorescence in situ hybridization.

A 1-year-old infant was referred to our hospital for gonadectomy and clitoroplasty. The patient was noted to have ambiguous genitalia with clitorimegaly soon after birth (Figure 1A and B). The right gonad was palpable in the labia majora, and the left one was not. The karyotype of peripheral lymphocytes was 46,XY and fluorescence in situ hybridization (FISH) for the *SRY* gene was positive. Ultrasound and magnetic resonance imaging studies revealed a hypoplastic uterus and an undescended gonad in the right hand side of the labia majora. At operation, the right gonad was a normal-appearing testis having the epididymis and deferent duct, whereas the left gonad resembled a streak gonad. Thus, gonadectomy was performed only for the right gonad. Histology of the gonad showed a streak testis consisting predominantly of differentiated normal-appearing seminiferous tubules with an area of undifferentiated gonadal tissue (UGT) and abnormally shaped seminiferous tubules at the periphery of the gonad (Figure 1C, Figure 2B–H). Nests of gonadoblastomas were present within the UGT (Figure 2C–E). The abnormally shaped seminiferous tubules consisted of focally proliferating germ cells, Sertoli cells, and pre-Sertoli/granulosa cells occasionally forming a cribriform arrangement, resembling a gonadoblastoma or an intratubular germ cell neoplasm (Figure 2F–H). Tissues derived from both Wolffian and Müllerian ducts were involved (Figure 1C). The clinical and histopathological characteristics were consistent with MGD.

### Methods

To confirm the diagnosis, we performed FISH for X and Y chromosomes and immunofluorescence for SRY along with testicular and ovarian lineage markers (SOX9 and FOXL2, respectively)^8,9^ to determine whether 45,X cells were included in the gonadal tissue. Written informed consent was obtained from parents for the biochemical and molecular studies, which were approved by the ethical committee of Tokyo Metropolitan Children's Medical Center.

Four-μm-thick sections were prepared from formalin-fixed paraffin-embedded tissues from the right gonad of this patient and a testis with a 46,XY karyotype was used as a control for FISH. Three normal testes with a 46,XY karyotype (2 weeks, 1 year, and 4 years old) were used as controls for immunohistochemistry. The methods for FISH and immunofluorescence are described in the supplemental material, http://links.lww.com/MD/A238.

Since FISH analysis was performed on thin paraffin sections, some cells with a 45,X appearance might have been false-negative artifacts.^5,10^ To exclude the possibility of such false-negative results, we used a digital image analyzer (WinRoof, Mitani Corp, Tokyo, Japan). This program counts the signals of chromosomes X and Y at a tissue level rather than cell by cell. The ratio of X to Y signals should be 1 if the tissue consists only of 46,XY cells, and 2 if half the cells were 45,X. Areas consisting of normal seminiferous tubules, UGT, and gonadoblastoma were photographed at a ×400 magnification, and fluorescence signals for the chromosome X and Y probes were counted in 10 different areas. This process was repeated by 2 of the authors (NN-U and RF).

To calculate the percentage of cells with 45,X, we rewrote the equation “R = 100/100-percentage mosaicism” in the following form: percent mosaicism = (R − 1)/R × 100, where R is the ratio of the number of the sex chromosomes (X chromosome/Y chromosome ratio) in the examined samples.

## RESULTS

### FISH Analysis of the Gonadal Tissue

FISH showed cells with both X and Y signals and cells only with an X signal, indicating that the gonadal tissue was composed of an admixture of 45,X and 46,XY cells (Figure 2A). On manual counting from photographs, the percentage of 45,X cells in our case was 44.6% (322/722, RF) and 56.4% (309/548, NN-U; average 49.7%), while that in the 46,XY control testis was 14.0% (48/343, RF) and 24.7% (84/340, NN-U; average 19.3%), which were significantly different (χ^2^ test, *P* < 0.001). However, this manual method had high inter-individual variation. To improve the precision, the percentage of 45,X cells was assessed by using the digital image analysis as follows. In the 46,XY control testis sample, the mean ratio of X and Y signals was 1.06 (the number of the X positive cells and Y positive cells was 1836 and 1735 per 10 areas [RF], 1864 and 1760 per 10 areas [NN-U]). These values were applied to the abovementioned formula, and the percentage of cells in the control negative for Y-chromosome (false negative) was 5.7%. In our case, the number of X-positive cells was much greater than that of Y-positive cells: the ratios of X and Y signals were 1.60 and 1.67, respectively (the numbers of the X-positive cells and Y-positive cells were 901 and 570 per 10 areas [RF], 1018 and 611 per 10 areas [NN-U]). Accordingly, the percentage of 45,X cells was 39.0% (37.5% and 40.1%, respectively), which was statistically different from that of the 46,XY normal control (5.7%; χ^2^ test, *P* < 0.001).

### Immunofluorescence for SOX9, FOXL2, and SRY in the Gonadal Tissue

**FIGURE 3:**
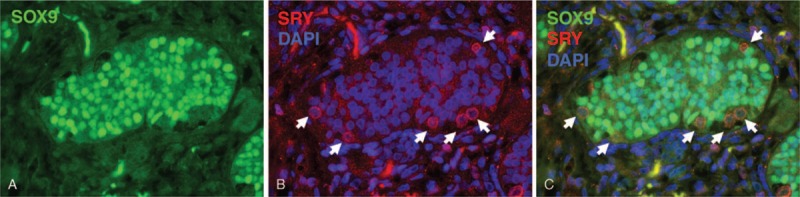
Immunofluorescence for SOX9 and SRY in a normal testis. (A) SOX9 is localized in the nuclei of Sertoli cells (green nuclei). (B) SRY is expressed in the cell membrane and cytoplasm of germ cells (red, indicated by arrows) and its expression is absent in nuclei (blue, DAPI). (C) Merged SOX9 with SRY (A) and DAPI (B). DAPI = 4′,6-diamidino-2-phenylindole.

SRY expression was assessed to identify XY and X cells, and expression of SOX9 and FOXL2 was used to assess testicular and ovarian differentiation, respectively. In normal-appearing seminiferous tubules, SOX9 was present in the nuclei of Sertoli cells and FOXL2 was not detected (Figure 2B), consistent with the staining pattern of the normal seminiferous tubules. SRY showed membranous and cytoplasmic localization in germ cells (Figure 2B), consistent with previous reports that showed localization of SRY switches from the nucleus of Sertoli cells to the cell membrane of germ cells after birth.^11^ Similarly, germ cells in gonadoblastomas and abnormally shaped seminiferous tubules had membranous/cytoplasmic expression of SRY (Figure 2D, E, G, H). We tested 3 normal testes with a 46,XY karyotype (2 weeks, 1 year, and 4 years old). Double immunofluorescence for SRY and SOX9 of those testes showed the same staining pattern as that of the normal areas of this case (Figure 3A–C). From these results, SOX9-only-positive cells were considered to be mature Sertoli cells with a 46,XY karyotype even though expression of SRY is lost.

Based on the abovementioned results, we defined 3 immunophenotypes of the cellular lineages that reflect differentiation toward testicular and/or ovarian epithelium (Table 1):

1. Sertoli cells that only express SOX9 (Figure 2B, F, H), differentiating toward the testicular lineage and constituting normal and abnormally shaped seminiferous tubules;
2. Precursor granulosa (pre-granulosa) cells that only express FOXL2 (Figure 2C and E), differentiating toward the ovarian lineage and appearing in gonadoblastomas; and
3. precursor Sertoli/granulosa (pre-Sertoli/granulosa) cells that are the bisexually differentiated sex cord epithelial cells that express both SOX9 and FOXL2 (Figure 2C and F), present in gonadoblastomas and abnormally shaped seminiferous tubules.

**TABLE 1:**
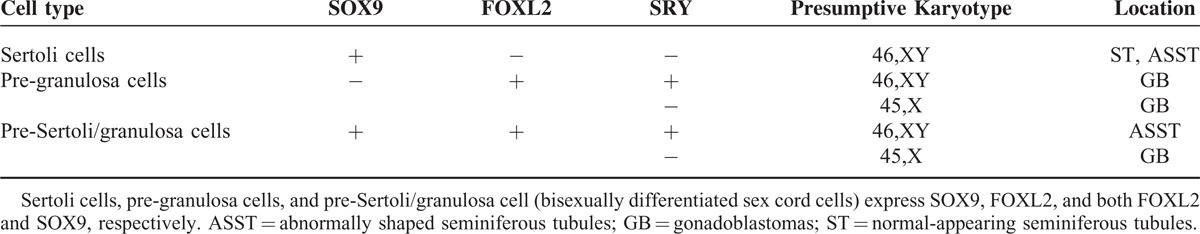
Immunophenotypes and Presumptive Karyotypes of Sex Cord Cells in Gonadoblastomas, Abnormally Shaped Seminiferous Tubules, and Normal-Appearing Seminifierous Tubules

The expression and localization of SRY were examined to estimate the presence or absence of Y-chromosome-containing pre-granulosa cells and pre-Sertoli/granulosa cells. Fluorescent signals for SRY were merged with those for SOX9 and FOXL2.

Sex cord epithelial cells in gonadoblastomas consisted mostly of pre-granulosa cells (Figure 2C). Almost all the FOXL2-expressing cells showed nuclear SRY positivity (Figure 2D). These results suggested that most sex cord epithelial cells constituting the gonadoblastoma are pre-granulosa cells with a 46,XY karyotype (sex reversal phenotype). However, there were a small number of pre-granulosa cells and pre-Sertoli/granulosa cells (Figure 2C) that lacked nuclear SRY signals (Figure 2D and E), confirming the presence of sex cord epithelial cells with a 45,X karyotype. The proportion of 45,X cells identified in immunofluorescence was approximately 10% (11/106 [RF], 9/87 [NN-U]), which was lower than that of 45,X cells identified in FISH. This discordance might be due to the inevitable occurrence of false negativity for the Y chromosome in FISH.

Sex cord epithelial cells in the abnormally shaped seminiferous tubules were composed of mature Sertoli cells (Figure 2F) and pre-Sertoli/granulosa cells (Figure 2F). All of the pre-Sertoli/granulosa cells also had nuclear SRY signals (Figure 2G and H). Sex cord epithelial cells with a 45,X karyotype were not evident. Stromal cells expressing SRY were also not apparent.

## DISCUSSION

In this study, we have confirmed that our patient had MGD by determining the 45,X/46,XY karyotype in the gonadal tissue using 2 different approaches: FISH and immunofluorescence analyses. We developed a digital image analysis program for counting X and Y chromosomes. Although it has been reported that approximately 24% of 45,X cells were observed in 46,XY normal testicular tissues,^5^ our method displayed reduced false negativity of only 5.7% of “45,X cells” in a normal testicular tissue with 46,XY. In combination with immunofluorescence for SRY, SOX9, and FOXL2, we could confidently identify 45,X cells in the gonadal tissue.

The gonadoblastomas were composed predominantly of pre-granulosa cells with 46,XY, and very few were 45,X cells. The majority of 45,X cells might undergo apoptosis and differentiate into stromal cells as is seen in the ovary (streak gonad) of Turner syndrome which is characterized by a 45,X or 45,X/46,XX karyotype. It is conceivable that since those stromal cells do not express SRY protein, the number of cells with 45,X detected by immunofluorescence appeared less compared with that detected by FISH.

Unexpectedly, our case showed co-expression of SOX9 and FOXL2 in abnormally shaped seminiferous tubules and gonadoblastomas. Hersmus et al^12^ described that SOX9 and FOXL2 were never “strongly” co-expressed in the same cell within the reasonably well-developed seminiferous tubules. We speculate that the level of FOXL2 expression in sex cord epithelial cells within seminiferous tubules might be variable between and within cases. As for gonadoblastoma, co-expression of SOX9 and FOXL2 was observed only in a minority of sex cord cells with 45,X. Lack of SRY might have caused dysregulation of SOX9 and FOXL2 expression.

FISH analysis on paraffin sections has the inevitable problem of false negativity for the Y chromosome. Immunohistochemical study for SRY is useful in proving the presence of Y chromosome; however, not all cell types express SRY protein and the interpretation of the results is complicated. Therefore, a combination of both the methods can be used to identify 45,X cells in the gonad of MGD patients with 46,XY.
